# SMAP Salinity Retrievals near the Sea-Ice Edge Using Multi-Channel AMSR2 Brightness Temperatures

**DOI:** 10.3390/rs13245120

**Published:** 2021-12-16

**Authors:** Thomas Meissner, Andrew Manaster

**Affiliations:** Remote Sensing Systems, 444 Tenth Street, Suite 200, Santa Rosa, CA 95401, USA

**Keywords:** SMAP, AMSR2, sea surface salinity, sea-ice, cold water

## Abstract

Sea-ice contamination in the antenna field of view constitutes a large error source in retrieving sea-surface salinity (SSS) with the spaceborne Soil Moisture Active Passive (SMAP) L-band radiometer. This is a major obstacle in the current NASA/Remote Sensing Systems (RSS) SMAP SSS retrieval algorithm in regards to obtaining accurate SSS measurements in the polar oceans. Our analysis finds a strong correlation between 8-day averaged SMAP L-band brightness temperature (TB) bias and TB measurements from the Advanced Microwave Scanning Radiometer (AMSR2) in the C-through Ka-band frequency range for sea-ice contaminated ocean scenes. We show how this correlation can be employed to develop: (1) a discriminant analysis that is able to reliably flag the SMAP observations for sea-ice contamination and (2) subsequently remove the sea-ice contamination from the SMAP observations, which results in significantly more accurate SMAP SSS retrievals near the sea-ice edge. We provide a case study that evaluates the performance of the proposed sea-ice flagging and correction algorithm. Our method is also able to detect drifting icebergs, which go often undetected in many available standard sea-ice products and thus result in spurious SMAP SSS retrievals.

## 1. Introduction

Three spaceborne L-band radiometers have been employed to measure sea-surface salinity (SSS) over the global ocean: (1) ESA’s Soil Moisture and Ocean Salinity (SMOS) mission, which has been operating since 2009; (2) NASA’s Aquarius mission, which operated between 2011 and 2015; and (3) NASA’s Soil Moisture Active Passive (SMAP) mission, which has been operating since 2015. These instruments measure the electromagnetic passive microwave emission from the Earth’s surface and atmosphere at a frequency of 1.41 GHz or wavelength of 21 cm. Retrieval algorithms for these sensors, which turn brightness temperature (TB) measurements into SSS, have been developed and successively refined [1]. Over open ocean scenes, it has been possible to achieve an average global accuracy of 0.2 psu or better for the retrieved SSS on monthly time scales and for 100-km spatial averages [2].

The retrieval accuracies of the L-band sensors strongly degrade in polar oceans. A major factor in this degradation is the decreased sensitivity of the L-band surface emission to SSS measurements at low sea-surface temperatures (SST). This results in a decreased signal-to-noise ratio in cold water (SST < 10 °C). To be specific, at colder SSTs, an error of +1 K in the TB measurement translates into an error of about –4 psu in the SSS retrieval. As such, improving SSS retrievals in or near polar regions has been identified as a major future science goal in ocean microwave remote sensing [3–6].

In this context, SSS retrievals close to the sea-ice edge have turned out to be particularly challenging. Even very small sea-ice fractions in the antenna field of view result in large spurious salinity biases. The degree of sea-ice contamination in the antenna field of view can be characterized by the quantity g_ice_, which is defined as the sea-ice concentration weighted by the antenna gain pattern [7]:

(1) $${\text{g}}_{\text{ice }}=\int \text{dAG}\cdot {\text{f}}_{\text{ice }}$$

here, G is the normalized antenna gain pattern projected on the Earth’s surface. The dA denotes the differential surface area, and f_ice_ is the sea-ice concentration (SIC) at the Earth’s surface. The integration in Equation (1) stretches over the whole Earth field of view. The measured emitted surface TB from the sea-ice contaminated Earth cell is given by:

(2) $${\text{T}}_{\text{B},\text{meas }}=\int \text{dAG}\cdot \left[\left(1-{\text{f}}_{\text{ice }}\right)\cdot {\text{T}}_{\text{B},\text{ocean }}+{\text{f}}_{\text{ice}}\cdot {\text{T}}_{\text{B},\text{ice}}\right]=\langle {\text{T}}_{\text{B},\text{ocean}}\rangle +{\text{ΔT}}_{\text{B},\text{c}}$$

with

(3) $${\text{ΔT}}_{\text{B},\text{c}}\approx {\text{g}}_{\text{ice }}\cdot \left[\langle {\text{T}}_{\text{B},\text{ice }}\rangle -\langle {\text{T}}_{\text{B},\text{ocean }}\rangle \right]$$

in Equation (3), we have assumed constant average values for ocean and sea-ice TB within the field of view that contributes to the integral. The term ΔT_B,c_ in Equation (3) can be regarded as a measure of sea-ice contamination in the measured TB. The TB of typical thicker sea-ice and ocean scenes differ by about 125 K for vertical polarization (V-pol). For horizontal polarization (H-pol) the sea-ice–ocean TB difference is even larger. We realize that an antenna gain weighted sea-ice fraction g_ice_ of only 1% will result in a positive TB bias error of about +1.25 K, which, in turn, translates into a fresh bias error of about –5 psu in the SSS retrieval. At L-band frequencies, the sea-ice TB depends on ice-thickness, which can vary over location and season, in particular in the N hemisphere [8]. It introduces further variability into the problem, which can be an additional source of error. All of this demonstrates the challenge of measuring SSS if even small sea-ice contamination occurs within the field of view. We also note that sea-ice contamination can occur through the antenna sidelobes, and, thus, the value for g_ice_ in Equation (3) can be sizeable, even if the SIC f_ice_ at the Earth boresight or within the main beam is zero. For both the Aquarius and the SMAP sensors, a sizeable portion of the antenna gain pattern G falls onto the sidelobes.

This discussion demonstrates the necessity for (1) developing a reliable procedure to detect small sea-ice concentrations in the field of view of the L-band sensor antenna; and (2) developing a correction for it. The second task amounts to estimating the contamination ΔT_B,c_ in Equation (3) as accurately as possible and, in turn, removing it from the TB measurement in Equation (2). One major obstacle in this methodology is the fact that available ancillary SIC products are generally not accurate enough to be used for detecting and removing sea-ice contamination in the SMAP salinity observations. Most of these standard SIC products, for example [9] or [10], apply a weather filter in their retrievals to screen out weather systems (clouds, rain, high winds), which also removes most but not all of the observations whose SIC is below 15%. To avoid sea-ice contamination in the SSS retrievals, the current NASA/RSS Version 4 SMAP Salinity release [11] employs a conservative ice mask, which results in a notable loss of data in the polar oceans [12].

**Figure 1.**
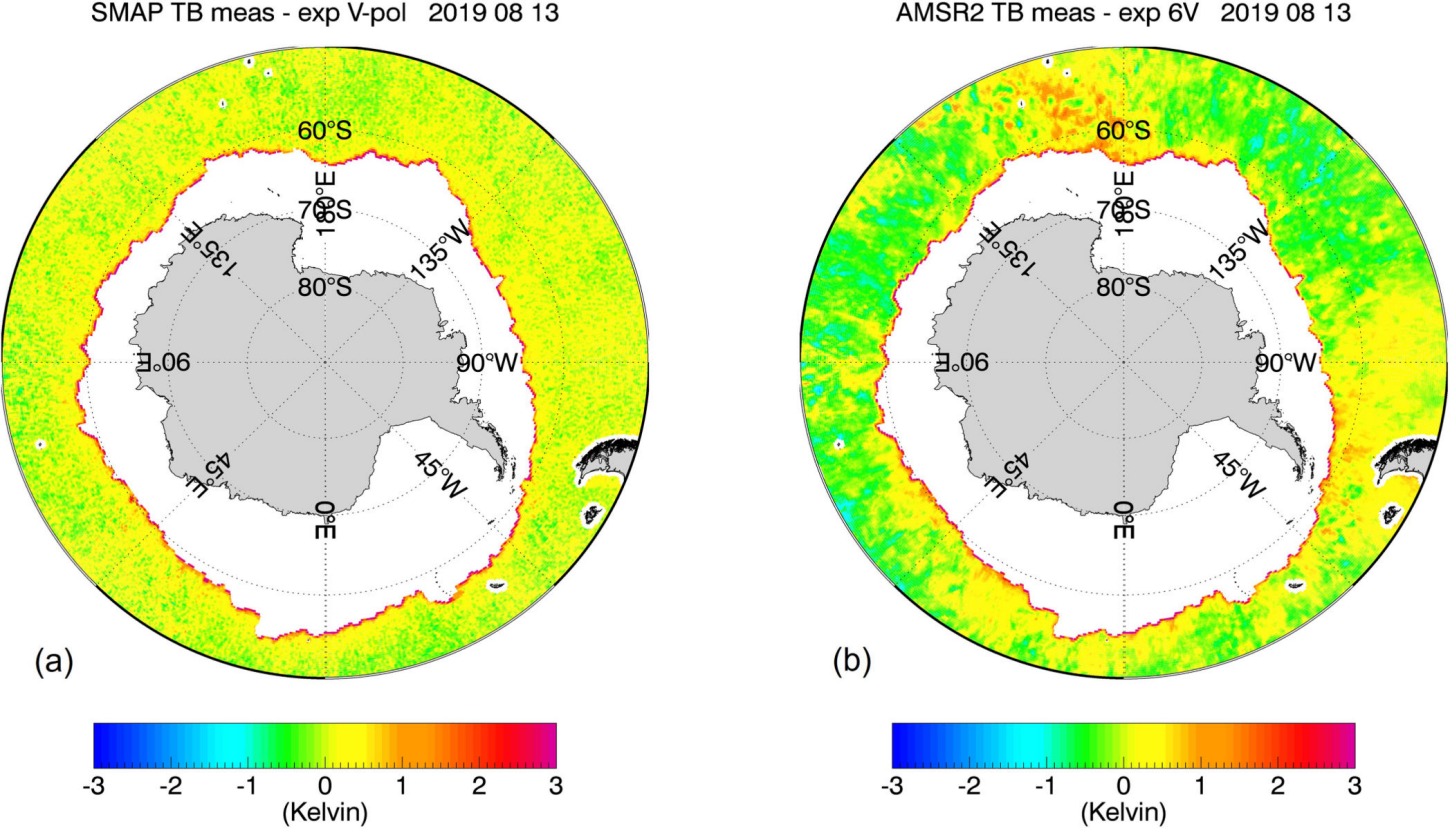
8-day average TB biases near the Antarctic sea-ice edge for a scene during the Austral winter (13 August 2019) (**a**) SMAP V-pol; (**b**) AMSR2 6.93 GHz V-pol channel. The TB bias is defined as the difference between measured and expected (computed) specular surface TB. The sea-ice contamination results in a positive TB bias for both SMAP and AMSR2 near the ice edge, which results in a fresh (negative) bias in the retrieved SSS.

**Figure 2.**
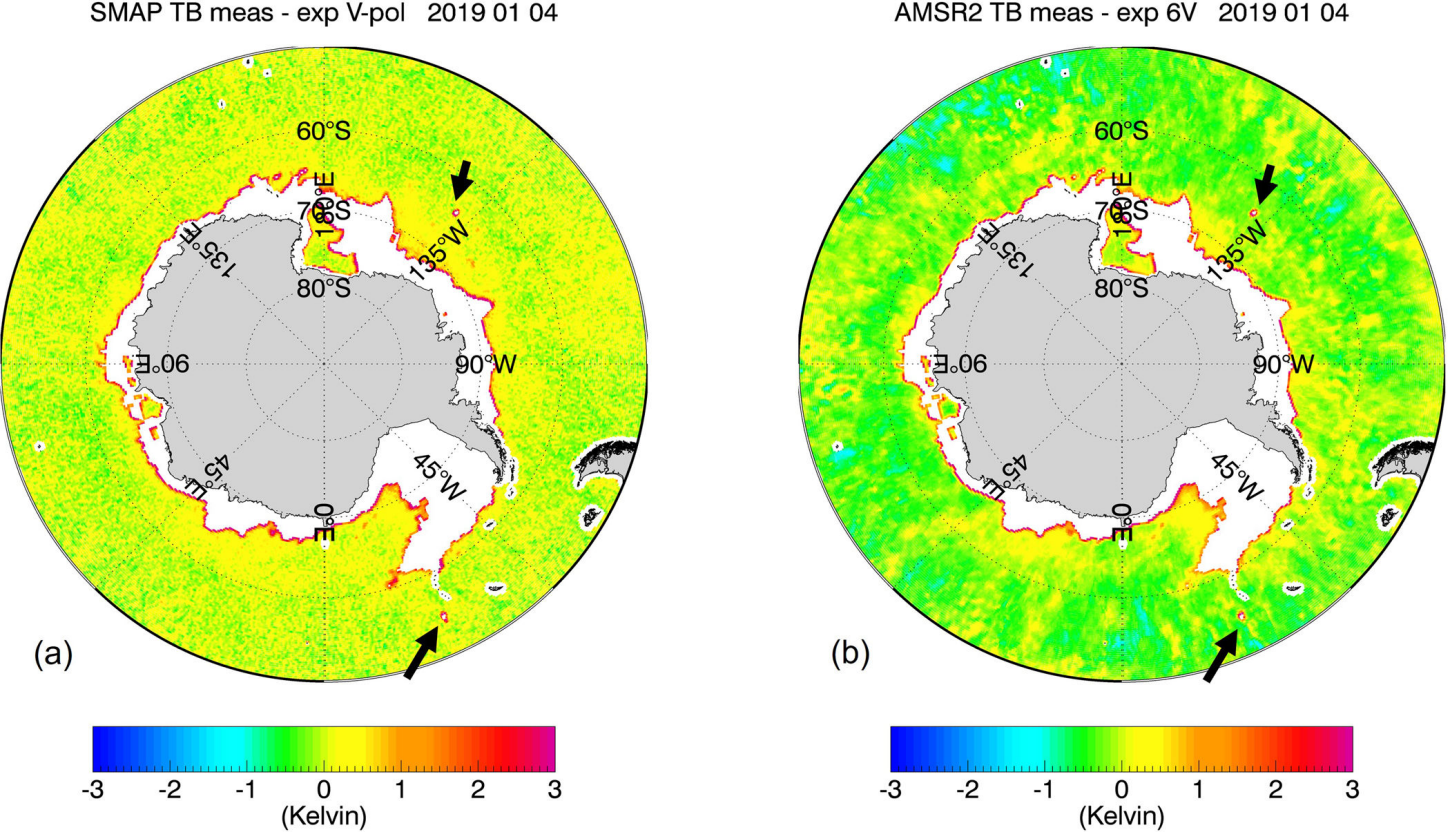
8-day average TB biases near the Antarctic sea-ice edge for a scene during the Austral summer (4 January 2019) (**a**) SMAP V-pol; (**b**) AMSR2 6.93 GHz V-pol channel. The TB bias is defined as the difference between measured and expected (computed) specular surface TB. The sea-ice contamination results in a positive TB bias for both SMAP and AMSR2 near the ice edge, which results in a fresh (negative) bias in the retrieved SSS. The black arrows indicate possible candidates of very large floating icebergs (Section 3.5).

The purpose of our study is to develop, train, and test sea-ice flagging and sea-ice correction algorithms for the SMAP SSS retrievals that can be implemented in future version updates. Our methods will not use external ancillary SIC fields due to their aforementioned insufficient accuracy in regards to low SIC near the sea-ice edge. Instead, both the SMAP sea-ice flagging and the sea-ice correction algorithms will use TB measurements from the JAXA Advanced Microwave Scanning Radiometer (AMSR2), which takes measurements at frequencies between C-band and W-band and both vertical (V-pol) and horizontal (H-pol) polarizations. This idea was inspired by Figures 1 and 2, which compare the eight-day averaged TB biases of the SMAP vertical polarization (V-pol) channel and the AMSR2 C-band (6.93 GHz) near the Antarctic sea-ice edge for two different periods during winter and summer seasons. The TB biases are determined as the difference between the measured TB at the ocean surface and the TB value computed from a radiative transfer model (RTM). We will give a more detailed account of the TB calculation in the next section. Visually, there is a strong correlation between these two TB biases near the ice edge. This suggests that there is the potential for the AMSR2 TBs to be directly integrated into the SMAP SSS retrieval algorithms for sea-ice flagging and correction. This is further supported by two key facts:

1. The spatial resolution of the SMAP footprint (≈40 km) and the lowest frequency AMSR2 frequency channel at C-band (≈50 km) are close. When multi-day aggregates are considered, in which several footprints are averaged together, then both sensors, SMAP and AMSR2, see approximately the same Earth scenes. Observations at the higher AMSR2 frequencies can be spatially resampled to the coarser C-band resolution.
2. Compared to the SMAP L-band channels, the AMSR2 channels are insensitive or only very weakly sensitive to SSS. The AMSR2 ocean measurements are affected by sea-ice contamination but not by signals such as freshwater river plumes or melt run-off. This enables us to separate the spurious sea-ice signal from the real freshwater signals.

The proposed sea-ice flagging algorithm is designed to detect sea-ice concentrations g_ice_ in the SMAP 40-km footprint of about 1.5% or more. This is a much lower detection threshold than the sea-ice edge, which is commonly defined as the location where the SIC is 15%. The low detection threshold is mandated by the aforementioned high sensitivity of the SSS retrieval to sea-ice contamination. On the other hand, it is also essential to keep the false alarm rate as low as possible. False alarms will either result in unnecessary data loss or overcorrection. It is quite evident in Figures 1 and 2 that the good correlation between SMAP and AMSR2 TB biases really only applies near the sea-ice edge and breaks down over the open ocean. Thus, applying a correction to observations that are not contaminated by sea-ice would introduce an unnecessary error in the SMAP SSS retrievals. The sea-ice detection algorithm is based on a discriminant analysis technique adapted from pattern recognition theory, which is designed to discriminate two different classes of data: uncontaminated open ocean scenes and sea-ice contaminated scenes. The discriminator analysis flag is then followed by additionally flagging the nearest neighbor (NN) to a given observation, which will extend the area of flagged observations. The NN flagging is necessary to catch SMAP observations that contain sea-ice contamination in the antenna sidelobes. The sea-ice correction algorithm is a statistical regression designed to recover as many of the flagged SMAP SSS observations as possible. Both, the sea-ice flagging and the sea-ice correction algorithm input AMSR2 TB together with several external ancillary geophysical fields. The use of these ancillary fields will increase the performance of the algorithm.

The vast majority of the scientific data users of the NASA/RSS SMAP SSS data utilize the eight-day or monthly time-averaged product from the Level 3 files. The Level 2 data are too noisy for most science applications. We have therefore decided to optimize our sea-ice flagging and sea-ice correction algorithms to perform best for the eight-day time-averaged SSS products. Thus, the analysis presented herein will focus on eight-day aggregate SMAP and AMSR2 TB observations.

In our study, we will present results for the SMAP observations at the 40-km sensor resolution. The standard NASA/RSS SMAP SSS product has been smoothed to a spatial resolution of 70 km, which results in a noise reduction over open ocean scenes that makes the product more useful for most scientific applications [13]. In regards to the evaluation of biases due to sea-ice contamination and the bias mitigation after the sea-ice correction, there is, unfortunately, little error reduction gained when spatially smoothing the product from 40 km to 70 km. Thus, it is appropriate to perform the analysis at the 40-km spatial scale.

## 2. Materials and Methods

### 2.1. Study Data Sets

#### 2.1.1. SMAP Brightness Temperatures

The source for the SMAP TB are the L2C files from the NASA/RSS SMAP SSS V4.0 Release [11], which have been sampled onto a 0.25° fixed Earth grid. For our study, we use the TB at the specular surface, labeled T_B0_(SMAP). It is calculated from the SMAP antenna temperature (TA) measurement after removing (1) antenna effects (antenna emissivity and cross-polarization contamination); (2) celestial radiation coming from the cosmic microwave background, galaxy, sun, and moon; (3) Faraday rotation in the Earth’s ionosphere; (4) atmospheric attenuation by oxygen, water vapor, and clouds; and (5) the wind induced rough surface emission [13–15]. We screen the observations for degrading conditions and discard data containing land contamination (land fraction > 1%), sun glint, large galactic radiation, rain, and very high wind speeds (>15 m/s). The L2C maps are then time-averaged into 8-day aggregate 0.25° SMAP T_B0_ maps.

#### 2.1.2 AMSR2 Brightness Temperatures

The source for the AMSR2 TB are the L1A AMSR2 TA measurements. Our study uses observations from the 6.93, 10.65, 18.7, 23.8, and 36.5 GHz V-pol and H-pol AMSR2 channels. We do not use the 2nd C-band channels (7.3 GHz) in our study as they contain no additional information to the 6.93 GHz channels in regard to developing our sea-ice flagging and correction algorithms. We also do not use the 89 GHz channels, as they are heavily affected by clouds and rain, which could introduce errors in our analysis. The observations of all channels are spatially resampled onto the largest footprint, which is one of the C-band (6.93 GHz) channels (62 km 35 km) [16]. The spatial resampling is based on the Backus-Gilbert optimum interpolation technique [17].

The AMSR2 TA are first transformed into the top of the atmosphere (TOA) TB, labeled T_B,TOA_(AMSR2), using the calibration procedure outlined in [18]. We employ the RTM to calculate the atmospheric attenuation [19] and the wind induced rough surface emission [20] for the AMSR2 channels to compute the specular surface TB for each channel, labeled T_B0_(AMSR2). In the calculation of the atmospheric attenuation, we use the simplified bulk expressions derived in [21] that depend on total columnar water vapor and total columnar cloud liquid water. The ancillary input fields to the RTM calculation will be discussed in Section 2.1.4. The specular surface emissivities E_0_(AMSR2) of the AMSR2 channels are determined by:

(4) $${\text{T}}_{\text{B}0}(\text{AMSR}2)={\text{E}}_{0}(\text{AMSR}2)\cdot {\text{T}}_{\text{S}}$$

where T_S_ is the SST in Kelvin.

The AMSR2 observations are screened for degraded conditions by removing measurements that are contaminated by land, sun-glint, known sources of radio frequency interference (RFI), and rain. When removing land contaminated measurements, we require that the land fraction within the AMSR2 6.93 GHz footprint is less than 1%.

Finally, the resampled AMSR2 TB are averaged in space and time into 8-day aggregate 0.25° maps, which comprise the same time periods as the SMAP TB map. When gridding the AMSR2 swath observations into regular 0.25° maps, we use a simple straight average (drop in the bucket). We will create and use separate sets of maps for the TOA T_B,TOA_(AMSR2), and the specular surface E_0_(AMSR2).

We note that the value of 1% for the land fraction threshold is the maximal land fraction that is allowed within a single AMSR2 C-band footprint. In each 0.25° cell several of these footprints are averaged together, so the actual land contamination is in general considerably less than 1% in the 0.25° cells, even at locations closest to the coast or near islands. The land fraction does not change over time, so by careful inspection of the cases used in this study we can check that land contamination is not a major error source in our analysis. We refer to the Supplementary Materials for maps that zoom into coastal areas and small islands.

#### 2.1.3. Expected Specular Surface Brightness Temperatures and Emissivities

The expected specular surface TB, labeled T_B0,exp_, is the result of the RTM calculation of the expected specular surface emissivity E_0,exp_ multiplied by T_S_. The expected surface emissivity E_0,exp_ is obtained from the Meissner–Wentz model of the dielectric constant (permittivity) of sea-water [15,20,22]. Our study calculates and uses expected T_B0,exp_ for SMAP, and expected E_0,exp_ for AMSR2.

The SMAP T_B0,exp_ are the basic metric for training and testing our proposed SMAP sea-ice flagging and sea-ice correction algorithms. At L-band frequencies, both T_B0,exp_ and E_0,exp_ depend on SST and SSS and, thus, their calculation requires an ancillary reference SSS field (see Section 2.1.4). The SMAP T_B0,exp_ are not an input for running the sea-ice flagging and sea-ice correction algorithms, but we use them only for developing the algorithms and evaluating their performances. Assuming that sea-ice contamination is the only uncertainty source in the SMAP SSS retrievals, then the difference between measured and expected SMAP specular surface TB is a proxy for the sea-ice contamination term ΔT_B,c_ in Equations (1) and (2):

(5) $${\text{ΔT}}_{\text{B},\text{c}}={\text{ΔT}}_{\text{B},0}(\text{SMAP})={\text{T}}_{\text{B}0,\text{ meas }}(\text{SMAP})-{\text{T}}_{\text{B}0,\exp}(\text{SMAP})$$

we have performed our analysis for both SMAP V-pol and H-pol measured–expected TB. All of the results turned out to be very close for both polarizations. Therefore, we will only present results for the SMAP V-pol channel for the remainder of the manuscript.

The AMSR2 E_0,exp_ will serve as actual input to the sea-ice flagging and sea-ice correction algorithms. In the C-through Ka-band frequency range of AMSR2, the E_0,exp_ do not depend or only depend very weakly on SSS. Thus, no external SSS input is required for their computation, and we can set the SSS in the dielectric constant model to a typical value of 35 psu without penalty. That is a crucial point for our proposed method.

As we did for the measured SMAP and AMSR2 TB, we also create time averaged 8-day aggregate 0.25° maps for the expected SMAP T_B0,exp_ and the AMSR2 E_0,exp_.

#### 2.1.4. External Ancillary Data

As noted earlier, our study requires various external ancillary fields. Specifically, we use the:

1. HYCOM SSS field [23] as the reference SSS. We are using the output from the operational ocean analysis that is run by the U.S. Navy [24]. In our study, this reference SSS is used in the computation of the SMAP T_B0,exp_, which serve as the basic metric for algorithm development and evaluation. The reference SSS field is not an input to the proposed sea-ice flagging or sea-ice correction algorithms or to the NASA/RSS SMAP SSS retrieval algorithm.
2. SST from the Canadian Meteorological Center (CMC) [25] as ancillary SST in the RTM computations for both SMAP and AMSR2.
3. Cross Calibrated Multi-Platform (CCMP) V2.0 wind speeds and directions [26–28] for the calculation of the wind induced rough surface emission for both SMAP and AMSR2. The CCMP winds are created by a variational analysis method that assimilates various microwave satellite wind measurements together with a background field from a numerical weather prediction model.
4. Atmospheric profiles for pressure, temperature, relative humidity, and cloud water density as well as total columnar water vapor and total columnar cloud water from the General Forecast System (GFS) of the National Centers for Environmental Prediction (NCEP) [29]. These fields are used for computing the atmospheric attenuation for both SMAP and AMSR2.
5. Precipitation rates from the Integrated Multi-satellitE Retrievals for the Global Precipitation Mission (IMERG) Version 6 [30] for rain-flagging of both SMAP and AMSR2 observations.

We note that these ancillary fields are the same that are already being used in the V4.0 NASA/RSS SMAP salinity retrieval algorithm for the purpose of turning measured SMAP TOA TB into surface emissivity. Extensive analysis has been conducted and shown that they are well suited for that purpose [14,31].

#### 2.1.5. Training and Test Sets

Our study is based on several training and test sets of SMAP and AMSR2 data, which are summarized in Table 1. For each of the two different years, 2018 and 2019, we have chosen one set during each of the 4 seasons (winter, spring, summer, fall) to accommodate a wide range of possible geophysical conditions near the ocean -sea-ice interface. Sets 1–4 from the year 2018 are used for training, and Sets 5–8 from the year 2019 are used for testing. That guarantees that training and testing data are independent. We use Data Set 9 from early 2021 for testing our algorithms for observations near floating icebergs.

As discussed in Section 2.1.4, we use the HYCOM SSS from the U.S. Navy operational ocean analysis as a reference field. The reference SSS is generally more reliable near the Antarctic sea-ice edge than near the Arctic sea-ice edge. In the Southern polar oceans, there exist reliable in-situ measurements from ARGO drifters, which serve as an important input to the operational ocean analysis data assimilation, whereas in the Northern polar oceans those in-situ measurements are sparse. Moreover, there are many freshwater river outflows into the Northern polar oceans, particularly around Northern Russia. In those instances, the reference SSS is often a poor representation of the true SSS field, which can result in a significant mismatch between measured and expected TB that is not caused by any sea-ice contamination. Last but not least, the L-band TB variability due to varying sea-ice thickness is much less prevalent in the Antarctic oceans than in the Arctic oceans, where large areas of thin sea-ice form near the sea-ice edge during fall. To avoid tainting the performance statistics, we will use only scenes in the Southern hemisphere for algorithm testing. We will show results for a case in the Arctic Ocean (Section 3.4) to demonstrate that the algorithm works there as well, but the observations from this case will not be included in the performance statistics.

The training of the algorithms turns out to be very robust and using scenes from the Northern hemisphere is not harmful. For training the sea-ice detection and flagging algorithm, we have found that it is actually slightly beneficial to use Arctic scenes, but only if the observations in known areas of freshwater river outflows are discarded.

#### 2.1.6. A-Priori Sea-Ice Flagging

**Figure 3.**
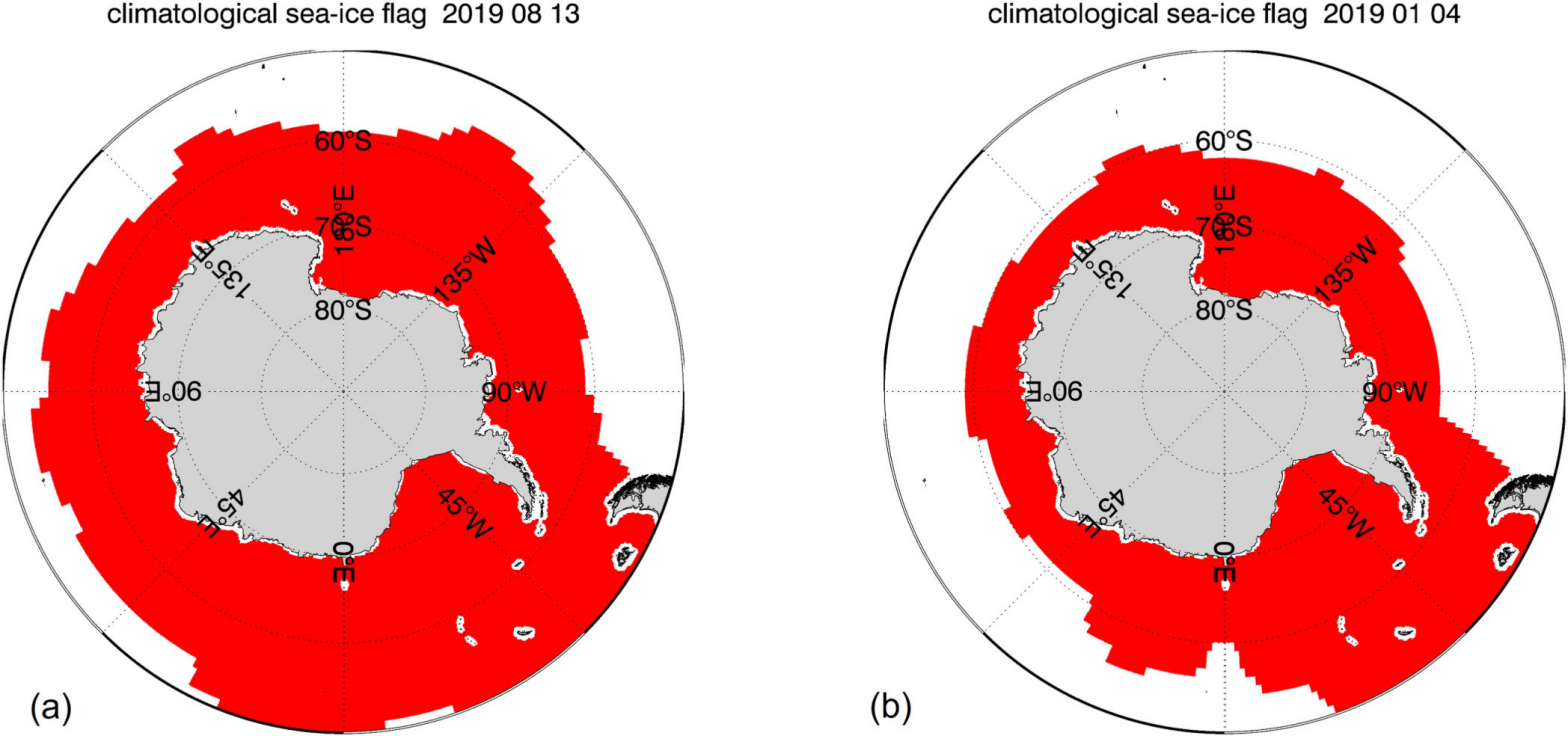
Climatological sea-ice masks near Antarctica for two sample days: (**a**) 13 August 2019 (Set 7-S); (**b**) 4 January 2019 (Set 5-S).

We impose a climatological monthly sea-ice mask as a-priori condition for the sea-ice detection and correction algorithms. This climatological sea-ice mask was compiled from many years of historical SIC data before the launch of SMAP and AMSR2. Its sole purpose is to provide a simple indicator for locations in the ocean where sea-ice can form. The mask also includes areas where drifting icebergs have been detected. Figure 3 shows the extent of the sea-ice mask for the example scenes from Figures 1 and 2. In addition, we also require the value of the ancillary SST to be lower than 10 °C. If either one of these two a-priori conditions are not met, the sea-ice detection algorithm is not run, the observation is not flagged as sea-ice, and no sea-ice correction is performed. The reason for applying these a-priori constraints is to avoid over-flagging and over-correcting observations that are very unlikely contaminated by sea-ice.

### 2.2. Sea-Ice Detection and Flagging

#### 2.2.1. Discriminant Analysis

The first and biggest step of the sea-ice detection and flagging algorithm employs the Fisher Discriminant Analysis, which is a well-established method in pattern classification for distinguishing two different classes of data points in a higher-dimensional space. Appendix A gives a summary and outline of this methodology.

When training the discriminant analysis, we draw data from two classes both of which are subsets of the 4 training sets listed in Table 1. These classes are defined as:

(6) $$\begin{array}{c} {\text{ΔT}}_{\text{B}0}{\left(\text{SMAP}\right)}_{\text{V}-\text{pol}}<{\text{e}}_{1}\Rightarrow \text{Class}\;1\\ {\text{e}}_{2}<{\text{ΔT}}_{\text{B}0}{\left(\text{SMAP}\right)}_{\text{V}-\text{pol}}<{\text{e}}_{3}\Rightarrow \text{Class}\;2 \end{array}$$

we have threshold values of e_1_ = 0.4 K, e_2_ = 2.0 K, e_3_ = 4.5 K. The RMS difference for 8-day aggregate ΔT_B0_(SMAP)_V-pol_ over open ocean scenes is found to be about 0.2 K, so any observation that falls within Class 2 is very likely contaminated by sea-ice. The sea-ice detection threshold e_2_ of 2.0 K corresponds to an approximate sea-ice concentration g_ice_ of 1.5%. If the discriminant analysis determines that an observation falls into this class, then it is flagged as sea-ice. Observations that fall within Class 1 might still contain some sea-ice contamination, and we address them in the second step of the sea-ice flagging algorithm. It is necessary to leave a large enough separation between the upper threshold e_1_ of Class 1 and the lower threshold e_2_ of Class 2. If the values for these thresholds are too close together, the discriminant analysis is less effectively able to distinguish between the two classes, which would result in an increased number of missed detections, false alarms, or both. The reason for imposing an upper threshold e_3_ in the definition of Class 2 will be discussed shortly.

The inputs to the discriminant analysis are the measurements **X** of the 10 AMSR2 TB channels, each of which represents a point in a 10-dimensional data space. We consider two cases for the AMSR2 data points **X**: Case 1 uses the TOA AMSR2 TB. Case 2 uses the measured–expected AMSR2 surface emissivities:

(7) $${\text{Case 1:}\;\text{X}}_{\text{k}}={\text{T}}_{\text{B},\text{TOA}}{\left(\text{AMSR}2\right)}_{\text{k}}\;{\text{ Case 2:}\;\text{X}}_{\text{k}}={\left[{\text{E}}_{0,\text{ meas }}(\text{ AMSR2 })-{\text{E}}_{0,\exp}(\text{ AMSR2 })\right]}_{\text{k}}\cdot {\text{T}}_{\text{eff }}$$

where the typical SST of T_eff_ = 273.15 K serves to convert the dimensionless emissivity values into quantities with dimension K. The index k in Equation (6) runs over the 10 AMSR2 channels.

**Figure 4.**
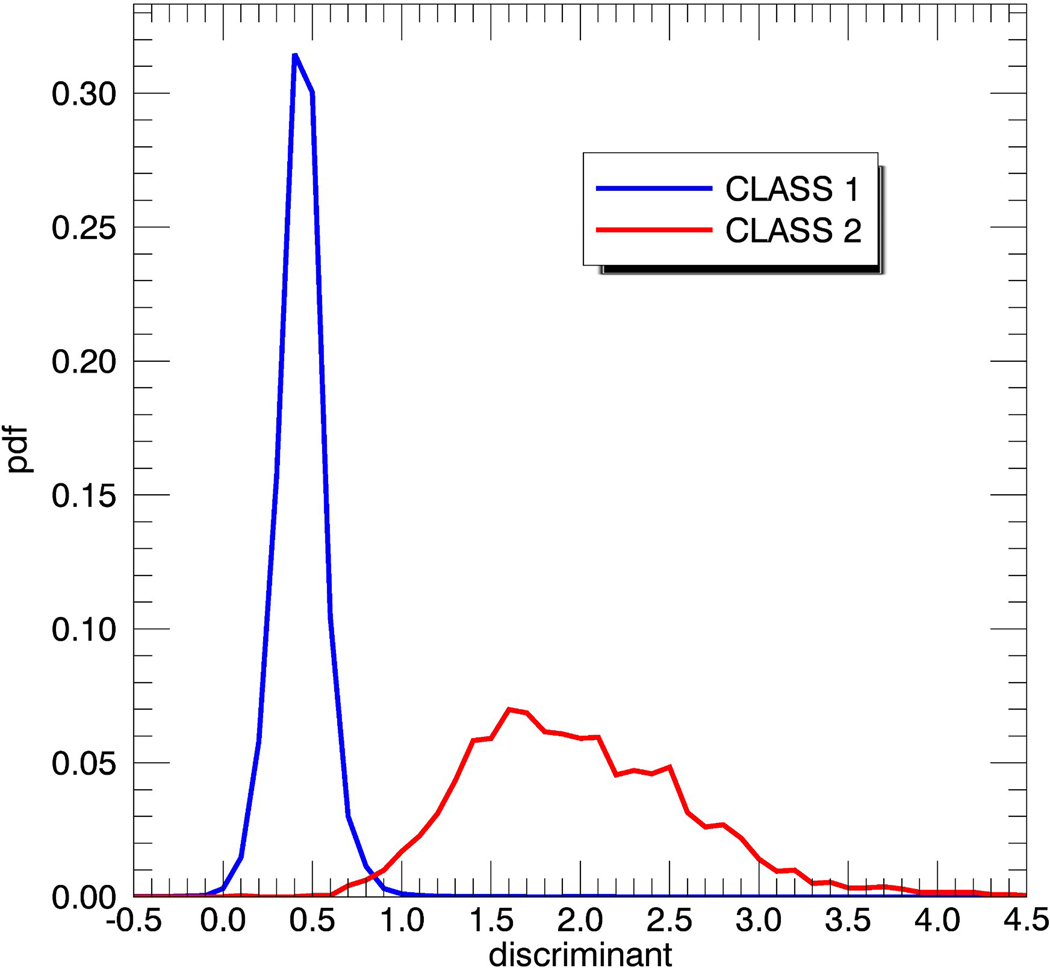
Probability density functions (pdf) of the two classes defined in Equation (5). The two pdf curves were computed for the training data sets (Table 1) and refer to case 2, which uses the AMSR2 measured minus expected specular surface emissivities as input. The *x*-axis values are the discriminant **W**^**T**^ · **X**. The decision boundary d is at the intersection between the two pdf, which in this example is d = 0.85.

The Fisher Linear Discriminant Analysis determines an optimal direction **W** within this 10-dimensional space, which results in the best separation between the two classes after the data are projected on it. The elements of the projection vector **W** can be regarded as optimal weighting coefficients of the 10 AMSR2 channels. Table 2 lists the results for the components of **W** that were determined from the training Sets 1–4 from Table 1 for both Case 1 and Case 2. We notice very low weights in the high-frequency channels, 23.8 and 36.5 GHz for Case 2, in which the atmospheric attenuation is explicitly removed. These higher frequency channels are more sensitive to the atmosphere and thus more impacted by uncertainties in the ancillary fields which are used for removing the atmospheric effects, in particular cloud liquid water and water vapor. The emissivity difference of the lower frequency AMSR2 channels, in particular C-band, correlates better with the observed SMAP ΔT_B0_. The discriminant is given by the projection of the data vector **X** onto the projection direction **W**, which is the scalar product **W**^**T**^ · **X** of the two vectors. For the determination of the criterion to distinguish between the two classes, we plot the probability density functions (PDFs) or histograms of the discriminant **W**^**T**^ · **X** for each class (Figure 4). The intersection point of the two PDFs defines the value d of the decision boundary and the decision criterion (A10) in Appendix A. The values for d for the two cases are also listed in Table 2. The values of **W** and d are determined from the training data sets and these values are subsequently used in the test runs.

Including an upper threshold value e_3_ in the condition for Class 2 in Equation (5) might look surprising at first. The reason for including this upper threshold is merely technical. For the numerical calculation of the optimal projection vector **W** from Equation (A9), it is desirable that the scatter matrices **S**_**i**_ in (A7) for both classes i = 1,2 have the same order of magnitude. This can be achieved by introducing an upper threshold in the definition of Class 2, as we have done. One strength of the Fisher Linear Discriminant Analysis is that it is robust when adding additional data points to Class 2. The detection algorithm has no difficulty placing any event for which ΔT_B0_(SMAP) exceeds the value e_3_ into Class 2, thus classifying it correctly as a sea-ice contaminated observation.

Table 3 lists the basic characteristics of this first step in the sea-ice detection algorithm, i.e., the missed detection and the false alarm rate. When calculating these values, we have only included observations that fall within the a-priori sea-ice mask (Section 2.1.6). That means that a rate of x% refers to x% from all observations that have been a-priori masked. We see that Case 2, which inputs AMSR2 specular surface emissivity, has better skill than Case 1, which inputs AMSR2 TB TOA, particularly in regard to false alarms. The calculation of the AMSR2 specular emissivity from the TOA TB requires external ancillary input fields for atmosphere, wind speed, wind direction, and SST followed by an RTM calculation of the atmospheric attenuation and the wind induced rough surface emission, which are then both removed from the TOA TB. The ancillary fields contain valuable information and removing the atmospheric and roughness effects helps to improve the skill of the detection algorithm. The benefit of removing atmospheric and wind roughness effects on sea-ice detection and SIC retrievals has been demonstrated in several studies [32–34].

#### 2.2.2. Nearest Neighbor Flagging

The discriminant analysis used in the 1st step of the sea-ice detection algorithm is designed to safely detect sea-ice contaminations of 1.5% or more as part of Class 2. However, observations that the discriminant analysis does not put into Class 2 can still be contaminated by sea-ice and thus might need to be flagged as well. Lowering the threshold value e_2_ in the class definition (5) would achieve that, but it would also result in an increased number of false alarms, which is not desirable. In order to catch the majority of these low SIC cases, we add a NN flagging as a 2nd step of the sea-ice detection algorithm. The basic assumption is that most of the sea-ice contaminated observations that went undetected in the 1st step are in close spatial proximity to at least one of the events that have already been flagged. Our sea-ice detection flags both the nearest neighbors and the next to nearest neighbors of any cell that the discriminant analysis has put into Class 2. This amounts to flagging the 24 surrounding cells of the 0.25° grid together with any cell from Class 2.

### 2.3. Sea-Ice Zones

**Figure 5.**
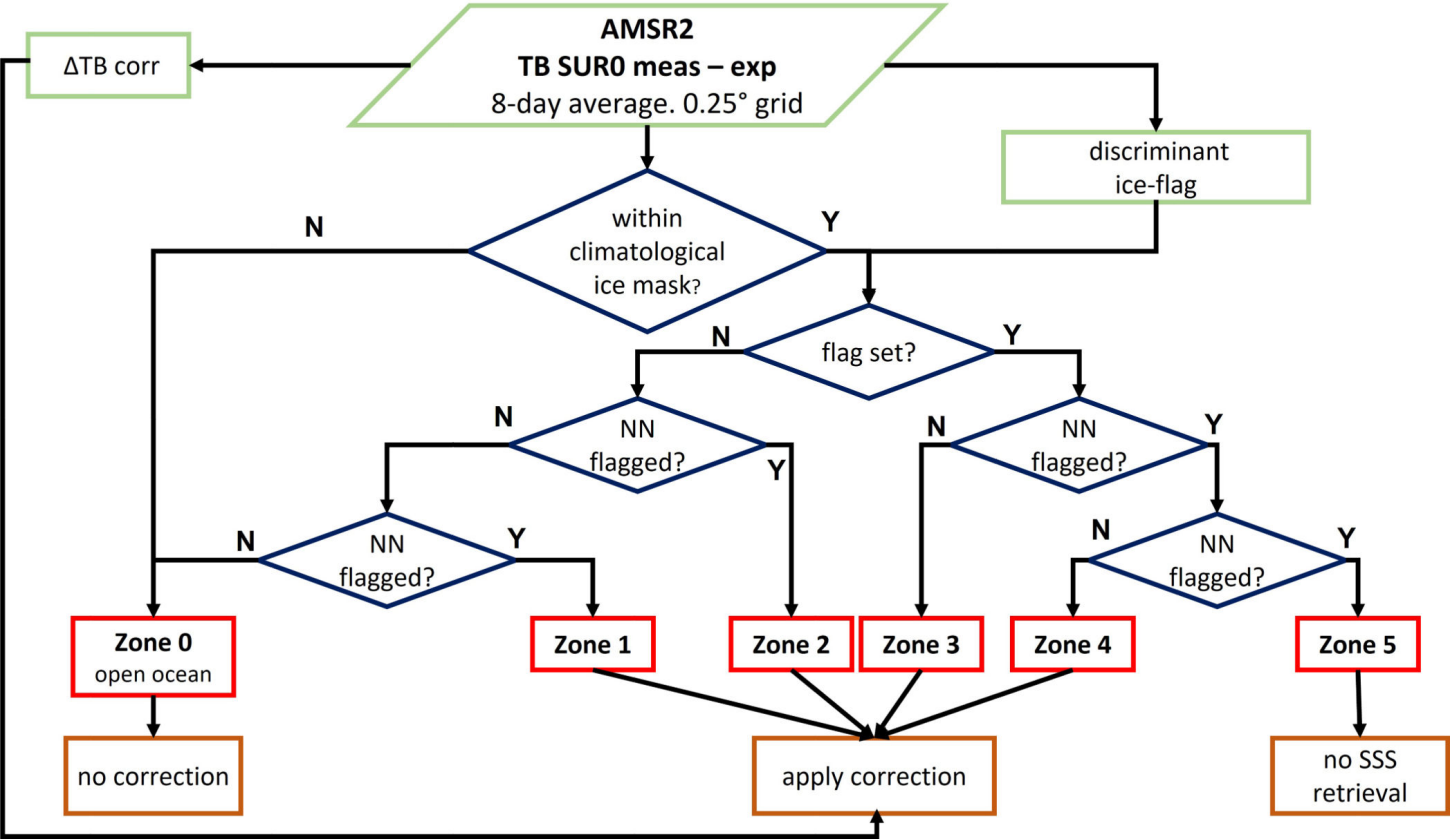
Schematic flow diagram of the sea-ice detection and flagging algorithm and defintion of the sea-ice zones 0–5.

**Figure 6.**
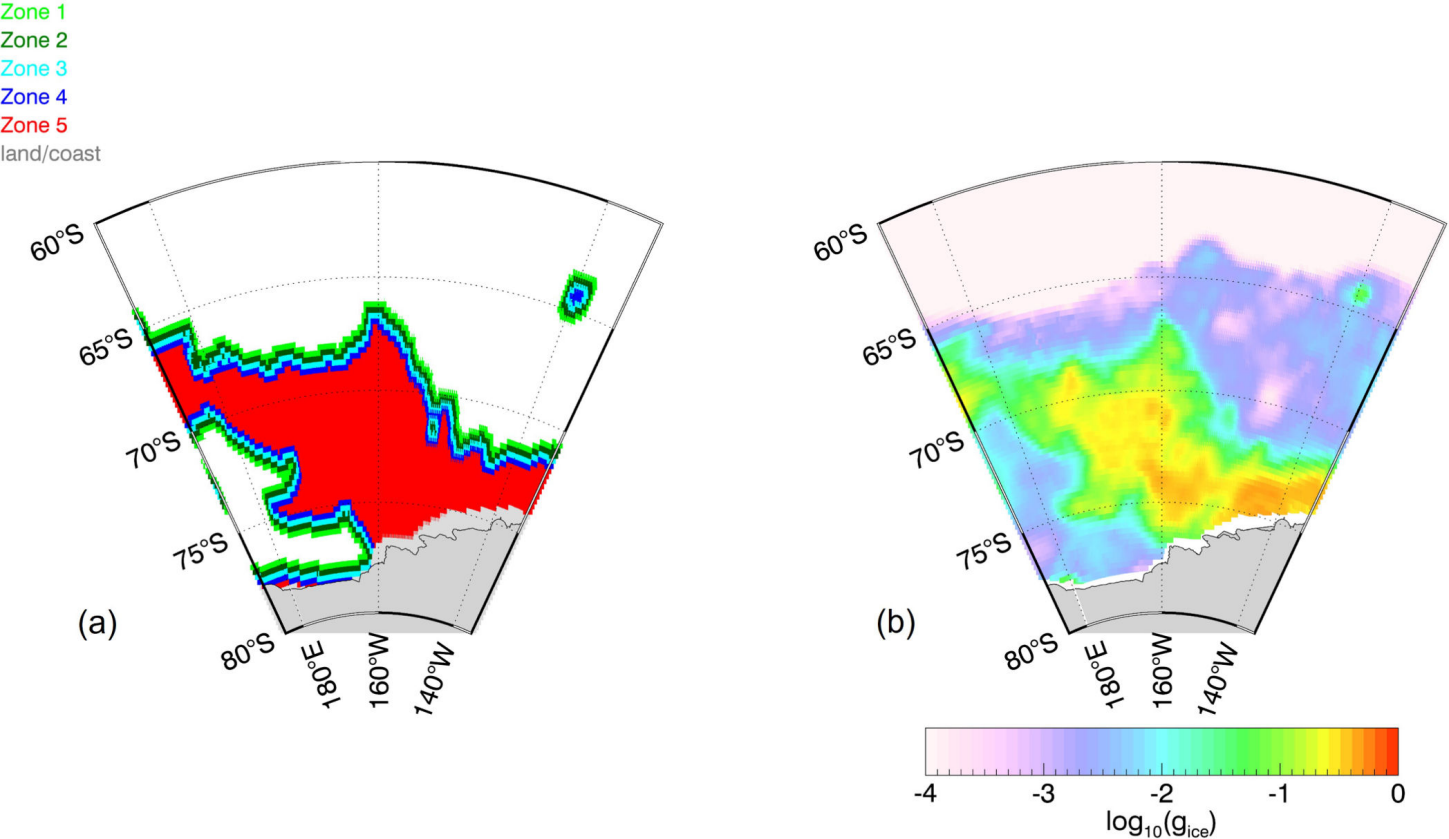
(**a**) Illustration of the sea-ice zones 0–5 for an area near the Antarctic ice shelf. Zone 0 (open ocean) observations are plotted white. The area near (65S, 140W) depicts a very large iceberg. (**b**) Antenna gain weighted sea-ice fraction g_ice_ from the OSI-SAF SIC product [10].

The combined discriminant and NN sea-ice flagging scheme allow us to define sea-ice zones, which classify the severity of sea-ice contamination for each observation. This will become helpful for developing and evaluating the sea-ice correction algorithm. Figure 5 shows the schematic flow and decision tree for sea-ice zone classification. An observation is placed into Zone 3 if it is flagged by the discriminant detection algorithm and at least one of its NN is not flagged. An observation that is flagged by the discriminant detection algorithm and at least one of its NN has been classified to be within Zone 3 is placed into Zone 4. The same decision is repeated for Zone 5 classification. An observation that has not been flagged by the discriminant algorithm but has at least one flagged NN, is placed within Zone 2. That decision is again repeated twice for classifying observations within Zone 1 and Zone 0. Observations that lie within Zone 0 are regarded as open ocean scenes. Figure 6a illustrates the five sea-ice zones for a sample scene near the Antarctic. It is evident that the distance to the denser sea-ice pack and thus the severity of sea-ice contamination increases when going from Zone 0 to Zone 5. For comparison, we have plotted the antenna gain weighted sea-ice fraction from the OSI-SAF SIC product [10] in Figure 6b. The sea-ice zones found by our algorithm match visually well with locations where g_ice_ (OSI-SAF) exceeds about 1%.

### 2.4. Sea-Ice Correction

The purpose of the sea-ice correction algorithm is to estimate the contamination term ΔT_B,c_ for the sea-ice flagged observations (given by Equations (2), (3) and (5)) and then subtract it from the measured SMAP T_B,0_ before the SSS is retrieved. As we did for the discriminant flagging algorithm, we again consider the two cases defined in Equation (6). Case 1 uses the TOA AMSR2 TB as input, and Case 2 uses the measured–expected AMSR2 surface emissivities as input. The corrections are determined as statistical linear regressions, which have the forms:

(8) $${\text{ΔT}}_{\text{B},\text{ corr }1}={\text{ΔT}}_{\text{B},0,1}(\text{SMAP})=\alpha_{0}+\sum_{\text{k}=1}^{10}\alpha_{\text{k}}\cdot {\text{T}}_{\text{B},\text{TOA}}{\left(\text{AMSR}2\right)}_{\text{k}}\;{\text{ΔT}}_{\text{B},\text{ corr2 }}={\text{ΔT}}_{\text{B},0,2}(\text{SMAP})=\sum_{\text{k}=1}^{10}\beta_{\text{k}}\cdot {\text{T}}_{\text{S}}\cdot {\left[{\text{E}}_{0,\text{ meas }}(\text{AMSR}2)-{\text{E}}_{0,\exp}(\text{AMSR}2)\right]}_{\text{k}}.$$

The indices 1 and 2 distinguish the two cases 1 and 2.

The regression coefficients αk and βk in Equation (7) are derived for the training set and then evaluated for the test set listed in Table 1. We derive separate regressions for each of the Zones 1–4. No sea-ice correction is performed for observations in Zone 0 (i.e., open ocean), in which sea-ice contamination is non-existent or small. Performing a correction, in this case, would likely introduce unnecessary errors into the SMAP SSS retrievals. Observations within Zone 5 are considered non-salvageable for the purpose of retrieving SSS.

We train and apply separate corrections for the SMAP V-pol and H-pol channels. Note that the regression for Case 2 in Equation (7) does not contain a constant term. The assumption is that the correction term should go to zero if the measured and the expected AMSR2 surface emissivities are the same, as in this case the g_ice_ goes to zero as well. We also note that sea-ice contamination always results in a positive TB bias. Because of noise in the observations, the calculated values of ΔT_B,c_ in Equation (7) are sometimes negative. In these instances, we set ΔT_B,corr_ = 0, and we do not apply a sea-ice correction.

## 3. Results

### 3.1. Correlation between SMAP and AMSR2 Brightness Temperatures

**Figure 7.**
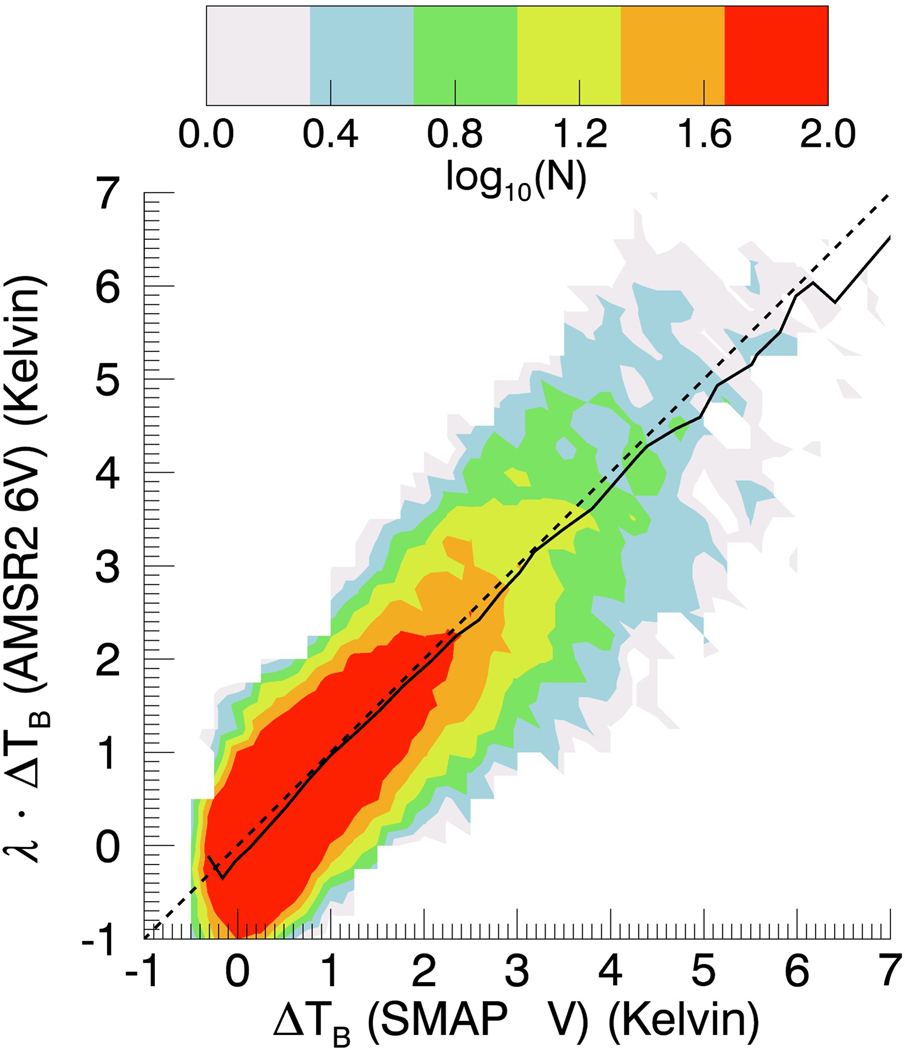
2-dimensional logarithmic joint histogram of the SMAP V-pol specular surface TB bias ΔT_B,0_(SMAP V) and the scaled AMSR2 6.93 GHz V-pol specular TB bias λ·Δ_TB,0_(AMSR2 6V) for the test set (Table 1) within sea-ice zones 1–4. The bin size is 0.25 K. The value of the scaling factor is λ = 1.15. The dashed line indicates the 1:1 line (ideal case). The full line indicates the binned average between *y*-axis versus *x*-axis and *x*-axis versus *y*-axis. The Pearson correlation coefficient is 0.96.

A pivotal point for the performance of the proposed flagging and correction method is the very good correlation between the SMAP and the AMSR2 measured–expected TB for the 8-day aggregate observations near the sea-ice edge. The ΔT_B,0_ of the two lowest AMSR2 frequency channels, 6.93 GHz and 10.65 GHz, correlate best with the SMAP ΔT_B,0._ This is mostly due to the fact that the higher AMSR2 frequencies are more strongly affected by atmospheric effects (i.e., water vapor and cloud water absorption), which act as noise sources in the detection and correction algorithms. Figure 7 shows a 2-dimensional logarithmic joint histogram of the SMAP V-pol specular surface TB bias ΔT_B,0_(SMAP V) and the scaled AMSR2 6.93 GHz V-pol specular TB bias λ ΔT_B,0_(AMSR2 6V) for the test set scenes (Table 1) within sea-ice zones 1–4. The scaling factor λ can be determined from a linear regression and is found to be λ = 1.15. The correlation coefficient is 0.96 (Table 4). Similar values are found (Table 4) for the correlation coefficients between the ΔT_B,0_(SMAP V) and the sea-ice correction terms ΔT_B,corr,1_ and ΔT_B,corr,2_ that were derived in Section 2.4. It is because of these high correlations, and the fact that the AMSR2 TB are insensitive to SSS, that we can employ them for sea-ice flagging and sea-ice correction in the SMAP SSS retrieval algorithm.

We note that the lowest frequency AMSR2 channels (C-band and X-bands) correlate best with SMAP, though the higher AMSR2 channels (Ku–Ka-bands) still contain valuable information when it comes to sea-ice contamination, and it is, therefore, beneficial to include them in our method.

### 3.2. Performance Evaluation of the Sea-Ice Correction Algorithm

Table 5 summarizes the performance evaluation statistics for the two cases of the sea-ice correction algorithm applied to the test cases within Zones 1–4 and compares them to the case without applying a correction. We have also included estimated values for the sea-ice fraction g_ice_ in each zone.

The correction algorithm was designed and trained to debias the SMAPΔT_B,0_ in each of the zones. The values show that this goal has been largely achieved. It is also encouraging to see reduced values for the standard deviations. The relative improvement compared to the uncorrected cases is strong for Zone 3 observations and even stronger for Zone 4 observations. We should note, however, that the remaining errors after the correction for Zone 3 and Zone 4 will still result in large errors in the SSS retrievals. A TB error of 1 K translates into an SSS error of about 4 psu in cold water (Section 1), and, thus, the expected SSS errors are about 5 psu in Zone 3 and about 12 psu in Zone 4. SSS errors of these magnitudes might still be too large for many scientific or operational oceanographic applications. Based on the results shown in Table 5, our analysis is able to give a reliable estimate of the SSS error in each of the zones. We expect that sea-ice corrected observations within Zone 1 and Zone 2 will be useful for the majority of scientific studies. The following sections will show several examples. Ultimately, it depends on the size of the signal of interest, and it will be up to the data users to decide if they want to discard the data for Zones 3 and 4.

Table 5 indicates that the Case 2 algorithm, which employs external ancillary fields and the RTM performs slightly better in Zones 1 and 2, whereas the Case 1 algorithm, which does not use external ancillary fields, performs slightly better in Zone 4. This is likely due to the fact that the quality of the external ancillary fields degrades with increasing sea-ice concentration. In particular, the ancillary SST and wind fields depend on satellite measurements, which become less accurate or are unavailable for observations within Zone 4. In Case 1, the higher frequency channels are indirectly employed to remove the atmospheric, wind, and SST effects from the TB TOA. In Case 2, this role is taken by the ancillary fields and the RTM calculation of atmospheric attenuation and wind induced rough surface emission. For Case 1, it is conceivable to develop a more elaborate machine learning technique, for instance, a neural network. It will need to be seen if this results in better performance than the statistical regression algorithm.

### 3.3. Antarctic Scene Examples

**Figure 8.**
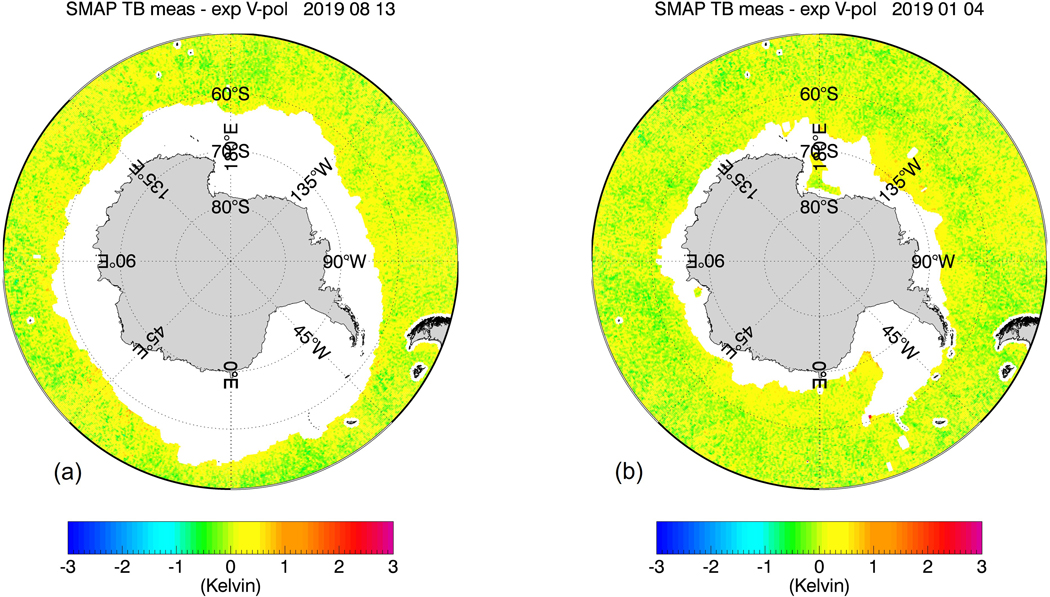
SMAP V-pol TB biases for the test cases from Figures 1 and 2 after discriminant and NN flagging. The resulting observations lie all within sea-ice Zone 0. (**a**) 13 August 2019; (**b**) 4 January 2019.

**Figure 9.**
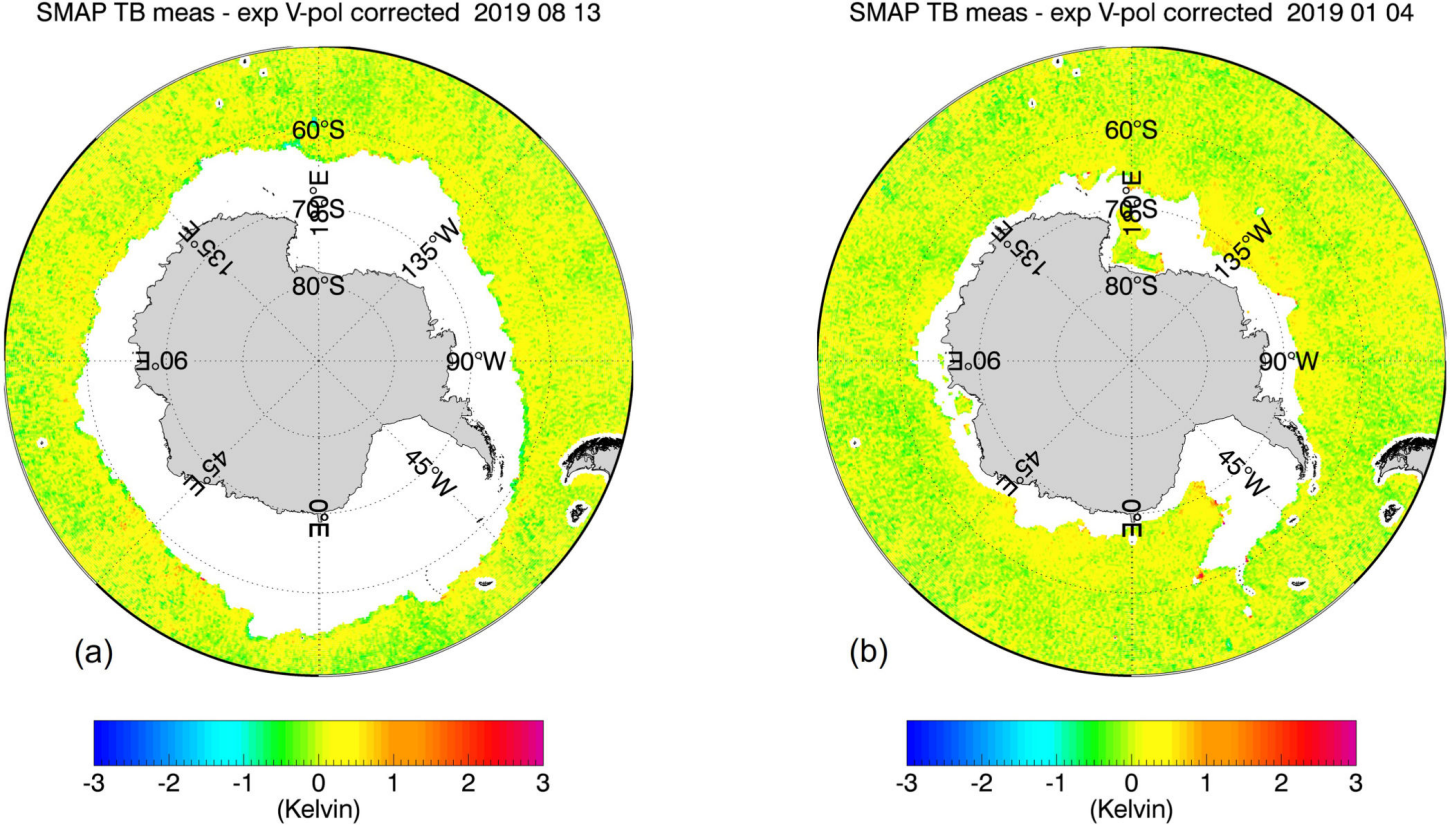
SMAP V-pol TB biases for the test cases from Figures 1 and 2 after flagging and sea-ice correction within sea-ice Zones 0–2. (**a**) 13 August 2019; (**b**) 4 January 2019.

Figure 8 shows the results of the sea-ice flagging algorithm when applied to the sample test scenes from Figures 1 and 2. The figure plots only observations that fall within Zone 0. This means that all of the sea-ice contaminated data have been removed resulting in almost bias-free scenes. Figure 9 shows the result within Zones 0–2 after the sea-ice correction algorithm has been applied. Only few cells are visible that are still contaminated by sea-ice. The vast majority of grid cells are free of large sea-ice contamination, and the SSS retrievals are of similar quality as over the open ocean. We will discuss the residual errors in ΔT_B,0_(SMAP) that remain after the sea-correction is applied in more detail in Section 4.

#### 3.4. Arctic Scene Example

**Figure 10.**
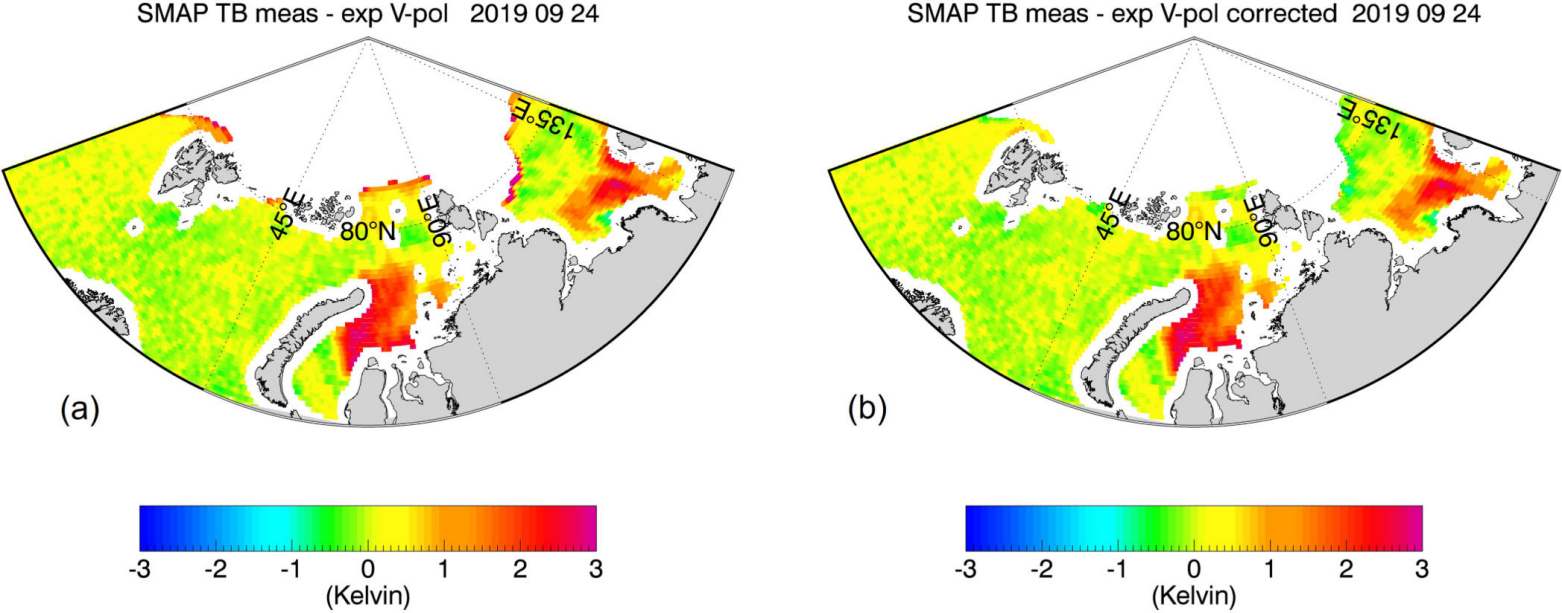
SMAP V-pol TB biases for an Arctic test scene on 24 September 2019 (Set 8 from Table 1). (**a**) before flagging and correction in zones 0–3; (**b**) after flagging and correction showing zones 0–2. The large red areas indicate freshwater river outflows. These areas are not affected by the sea-ice flagging and correction algorithms.

Figure 10 shows an example of how the algorithms perform in the Arctic. The large red areas are freshwater outflows from Siberian rivers. This positive SMAP TB bias is most likely caused by the HYCOM reference SSS underestimating the freshening caused by these river outflows whereas SMAP picks it up. AMSR2 is not sensitive to the freshening, and, thus, these areas remain unflagged, and no sea-ice correction is done. This is the desired outcome i.e., areas with no sea-ice should not be flagged as such. On the other hand, it is evident from Figure 10 that our algorithm is capable of detecting, flagging, and correcting the sea-ice contamination near the sea-ice edge, where spurious SMAP TB biases are observed.

### 3.5. Icebergs

Very large drifting icebergs are a potential source of serious errors in the SMAP SSS retrievals. Even though icebergs are not sea-ice, the electromagnetic properties of sea-ice and icebergs result in a similar degree of contamination in the SSS measurement. The a-priori climatological ice mask (Section 2.1.6) deliberately includes areas that are prone to contain drifting icebergs. That is essential to enable the sea-ice flagging algorithm to also detect icebergs. A closer look at Figure 2 shows some very large icebergs near Antarctica, which are both visible in the SMAP TB and in the AMSR2 6V TB. Checking the results of the sea-ice flagging algorithm (Figure 8b) and the sea-ice correction algorithm (Figure 9b) for the same scene indicates that our method is able to detect these icebergs and correct the contamination to a good extent.

**Figure 11.**
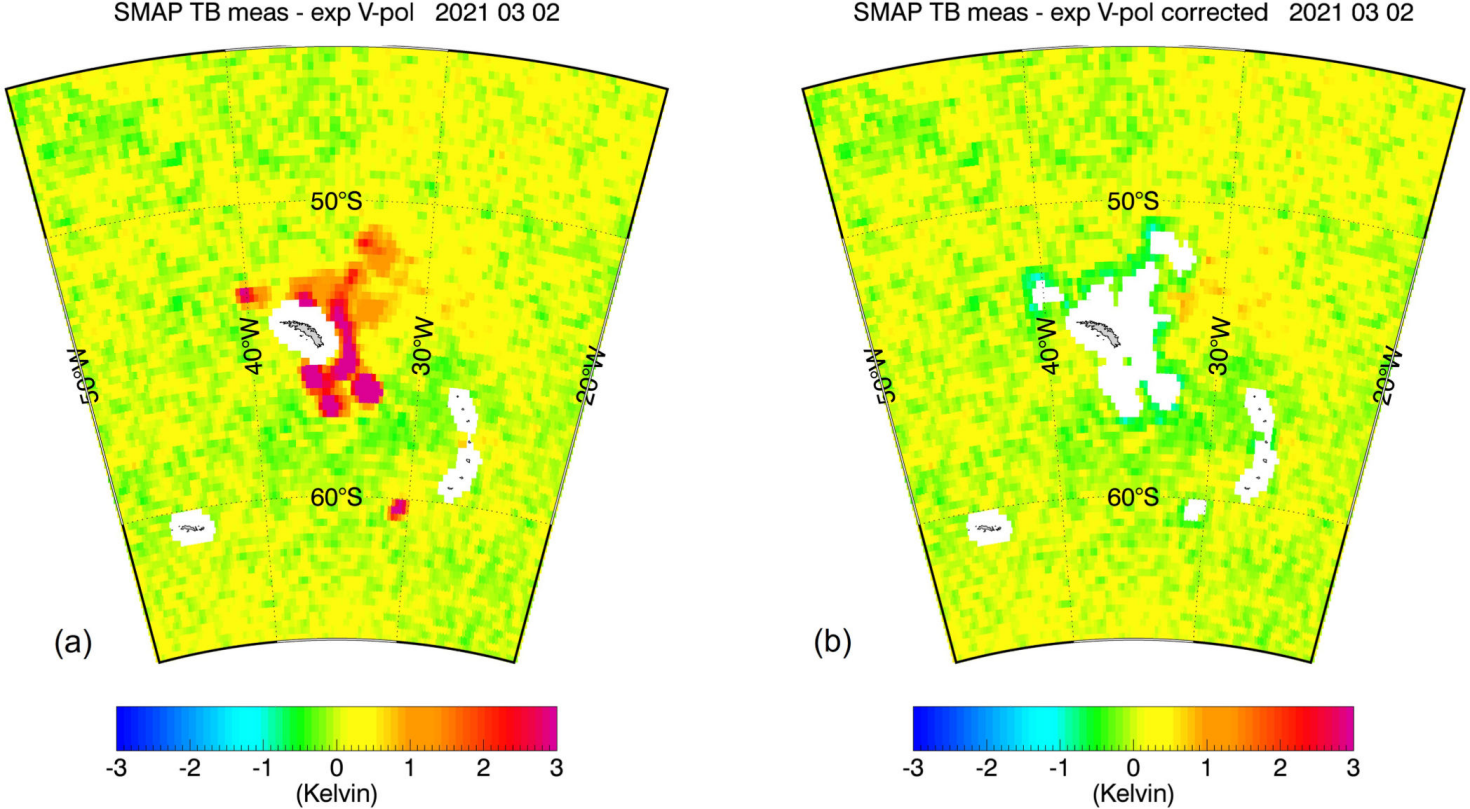
SMAP V-pol TB biases for an area around the South Georgia Islands near the Antarctica on 02 March 2021 (Set 9 from Table 1) that was affected by the very large drifting iceberg A-68 [35]. (**a**) before flagging and correction; (**b**) after flagging and correction showing zones 0–2.

A good example of this detection and correction is provided by the very large iceberg A-68 [35], which was drifting near the South Georgia Islands and several hundred kilometers away from the Antarctic sea-ice edge in the early months of 2021. It was the cause of large spurious fresh SMAP SSS values in that location, whose fresh biases exceeded 10 psu. This resulted in a surface area of about 250,000 km^2^ of undetected contaminated SMAP SSS data over the course of several weeks. Figure 11 shows (a) the iceberg contaminated SMAP data and (b) the result after flagging and correcting the contaminated observations. Our method appears to be very effective in detecting and mitigating the problem.

We note that neither of the products [9,10] shows this iceberg in their SIC data.

## 4. Discussion

This section discusses the uncertainty and error sources in our methodology and its limitations.

1. Radiometer noise in the AMSR2 TB measurements is a source of error. However, the noise in the individual measurements is strongly reduced in the eight-day averages. Thus, the impact of AMSR2 radiometer noise on our detection and correction algorithms is small.
2. A larger uncertainty source are errors in the external ancillary fields, which might not get suppressed in the eight-day averaging. This impacts the algorithms from Case 2, which use the ancillary fields to remove atmospheric and wind roughness effects from the AMSR2 TOA TB. We expect the errors in the ancillary fields for SST, wind speed, and direction to increase with increasing sea-ice fraction g_ice_ and decreasing distance from the sea-ice edge. The ancillary data sets for these parameters are Level 4 products that rely on microwave satellite observations as input. When approaching the ice edge, the microwave satellite observations are flagged, and, thus, fewer satellite input data become available. This can result in lower accuracy of the ancillary Level 4 product [26].
3. One of the major assumptions when using the AMSR2 TB to flag and correct the sea-ice contaminated SMAP TB was that the spatial resolutions of the SMAP and the AMSR2 TB measurements are similar. When averaged over eight days, the different orientations of the antenna footprints at a certain location have less of an impact. This means that SMAP and AMSR2 see approximately the same Earth scene. That assumption is of course only approximately fulfilled, and in reality, there is some spatial mismatch between the SMAP and the AMSR2 observations. It is possible to reduce this uncertainty source by resampling the SMAP and all the AMSR2 channels to the same target location and the same spatial resolution by employing the Backus-Gilbert optimum interpolation technique [17]. This resampling would use a polar stereographic projection for selecting the target locations, which is better suited than a regular 0.25° grid in polar regions. This is a project that is currently being considered, but it will require considerable effort.
4. There are scenarios that can result in a degradation of the good correlation between the SMAP and the AMSR2 TB. The most important one is thin sea-ice, which impacts the higher AMSR2 frequencies more than the lower ones or the SMAP L-band. We note that, at L-band, the sea-ice penetration depth is approximately 0.5 m, and the penetration depth grows with the electromagnetic wavelength. Large areas of thin ice tend to form in the Arctic oceans during fall. In the S hemisphere, this problem is less prevalent. Further studies are necessary to examine if and how much this poses a problem for our flagging and correction method.
5. Undetected RFI in the AMSR2 TB also constitutes an error source.
6. A problematic case is sea-ice close to the coast, which can result in simultaneous land and sea-ice contamination of the SMAP antenna field of view. Our proposed sea-ice detection and correction algorithms were not designed to handle this case. Likewise, we expect that the sidelobe correction for land contamination that is currently applied in the NASA/RSS SMAP SSS retrievals [13,14] is not able to cope with this scenario either.
7. The flagging method is not able to detect observations of very small sea-ice fractions that are significantly lower than the detection threshold of the discriminant analysis (1.5%) and that are not NN or next to NN of an observation that has been flagged by the discriminant analysis.
8. The statistical linear regressions that are used in the sea-ice correction algorithm are designed to de-bias the ΔT_B,0_(SMAP) in each sea-ice zone, which they do (Table 5). The residual errors after applying the correction approximately add to zero when summing over all test scenes with standard deviation values listed in Table 5. However, we note that the spatial correlation scales of these residual errors can be quite large. For instance, in Figures 9a, 10b, and 11b green bands are visible in the corrected observations, which indicate larger areas where the algorithm slightly overcorrects. In Figure 9b, a few yellow–orange areas are visible, which indicate that the algorithm undercorrects in these locations. That means that we should not expect that this residual error can be significantly reduced by spatial smoothing. Consequently, we anticipate that the remaining errors in the 70-km NASA/RSS SMAP SSS product will have a similar magnitude than in the 40-km product near the sea-ice edge.

## 5. Conclusions

The AMSR2 TB measurements between C-band and Ka-band contain valuable information that can be employed in flagging and correcting sea-ice contaminated SMAP observations. This will result in better SMAP SSS measurements in polar oceans that currently suffer from sea-ice contamination, which is otherwise difficult to detect and remove. We plan to implement both the sea-ice flagging and the sea-ice detection algorithm into the upcoming Version 5 release of the NASA/RSS SMAP SSS product. The calculated correction ΔT_B,corr_ (Equation (7)) allows us to provide an approximate value for the gain weighted sea-ice fraction g_ice_ in the SMAP antenna field of view and thus of the severity of the sea-ice contamination. The performance results from Table 5 can be used to estimate the residual RMS error in the retrieved SSS after the sea-ice correction is performed.

More extensive validation of the sea-ice corrected SSS retrieval is warranted and will be carried out. It will be particularly important to conduct a comparison with in-situ ground-truth measurements in the polar oceans, e.g., saildrones [36].

Possible future improvements or refinements include spatial resampling of the SMAP and AMSR2 observations to the same location and spatial resolution, as mentioned in Section 4. It is also desirable to see if the temporal resolution can be improved. We have designed the SMAP sea-ice detection and correction algorithms for eight-day time-averaged observations as our main goal was to improve the Level 3 eight-day SMAP SSS products, which are used in most scientific applications. However, sea-ice concentration and contamination can change within eight days. In particular, near the ice edge, sea-ice motion can exceed 100 km within this eight-day time window both along and across the ice edge. Our method is based on the time-averaged SIC at a certain location. This time-averaged SIC is approximately the same for the AMSR2 and the SMAP eight-day aggregates, as long as there are no temporal gaps in data acquisition for either of the two sensors. A shorter time window for the AMSR2 aggregates is warranted when dealing with Level 2 retrievals, which are of interest for most operational applications. That said, a smaller time window will increase the noise in the sea-ice detection and correction algorithms. We plan to explore if the time averaging window could be narrowed without increasing the missed detection and false alarm rates too much.

We expect that the proposed methodology could be applied to Aquarius SSS retrievals as well. This would require that the AMSR2 observations being spatially resampled to the lower Aquarius resolution of 100–150 km. Adapting this methodology to SMOS, which is a synthetic aperture radiometer, is more difficult and it is not clear if it is feasible. It is safe to assume that the method can be continued with AMSR3, which is planned to be launched in the 2023 time frame, if SMAP is still operating by then. A very exciting future mission that can apply our methodology is the European Copernicus Imaging Microwave Radiometer (CIMR) [37], which is planned to start operating by the end of this decade. CIMR will simultaneously observe the same Earth scene at a wide frequency range between L-band and Ka-band, and, thus, provide most of the necessary input for our methodology with one single instantaneous measurement.

## Supplementary Material

support data

## Figures and Tables

**Table 1.** Date and orbit ranges for the 8-day SMAP and AMSR2 TB data sets that are used for training and testing the sea-ice flagging and sea-ice correction algorithms.

set	date range	SMAP orbit range	AMSR2 orbit range	pole	used for
**1**	21 – 28 JAN 2018	15875 – 15991	30209 – 30325	N	training discriminant flagging
				S	training regressions for sea-ice correction
**2**	14 – 21 APR 2018	17089 – 17205	31418 – 31533	N	training discriminant flagging
				S	training regressions for sea-ice correction
**3**	16 – 23 JUL 2018	18448 – 18565	32772 – 32888	N	training discriminant flagging
				S	training regressions for sea-ice correction
**4**	09 – 16 OCT 2018	19692 – 19808	34010 – 34125	N	training discriminant flagging
				S	training regressions for sea-ice correction
**5**	01 – 08 JAN 2019	20920 – 21037	35233 – 35349	S	testing discriminant flagging testing regressions for sea-ice correction
**6**	24 – 31 MAR 2019	22120 – 22236	36427 – 36543	S	
**7**	10 – 17 AUG 2019	24153 – 24269	38452 – 38567	S	
**8**	21 – 28 SEP 2019	24767 – 24883	39063 – 39179	S	
				N	Arctic example (section 3.4.)
**9**	27 FEB – 06 MAR 2021	32445 – 32561	46708 – 46824	S	iceberg A-68

**Table 2.** Values of the components of the Fisher Linear Discriminant Analysis projection vector **W** (Appendix A) within the 10-dimensional space spanned by the AMSR2 TB channels in the training data set (Table 1). The length of the vector **W** has been normalized to 1. Case 1 refers to the algorithm using the AMSR2 TOA as input. Case 2 refers to the algorithm using the AMSR2 measured minus expected specular surface emissivities as input. The last column contains the values for the decision boundary d for each case.

channel i	6.93 V	6.93 H	10.65 V	10.65 H	18.7 V	18.7 H	23.8 V	23.8 H	36.5 V	36.5 H	d
**W_i_ (case 1)**	0.140082	−0.46514	0.254423	−0.08172	−0.62169	0.486014	0.168304	−0.12771	−0.15391	0.03985	52.05
**W_i_ (case 2)**	0.01366	−0.50493	0.43747	−0.10526	−0.70372	0.20662	−0.00025	0.06365	−0.00406	0.02058	0.85

**Table 3.** Missed detection and false alarm rate for the discriminant sea-ice flag in the test data set (Table 1). Only observations within the a-priori sea-ice flagging (section 2.1.6.) are considered for calculating the rates. Case 1 refers to the algorithm using the AMSR2 TOA as input. Case 2 refers to the algorithm using the AMSR2 measured minus expected specular surface emissivities as input. A missed detection is defined as an observation, which the detection algorithm places into Class 1 but for which ΔT_B,0_(SMAP) > e_2_ = 2.0 K. A false alarm is defined as an observation, which the detection algorithm places into Class 2 but for which ΔT_B,0_(SMAP) < e_1_ = 0.4 K.

	Case 1	Case 2
**missed detection rate**	0.15%	0.12%
**false alarm rate**	0.39%	0.06%

**Table 4.** Pearson correlation coefficients between the SMAP V-pol specular surface TB bias ΔT_B,0_(SMAP V) for the test set scenes (Table 1) within sea-ice zones 1 – 4. The ΔT_B,0_ (6V) is the AMSR2 6.93 GHz V-pol specular TB bias. The ΔT_B,corr1_ and ΔT_B,corr2_ are the values for the sea-ice corrections that were derived in Section 2.4.

product	ΔT_B,0_ (6V)	ΔT_B,corr1_	ΔT_B,corr2_
**correlation**	0.96	0.96	0.96

**Table 5.** Performance statistics of the sea-ice correction algorithm (Section 2.4.) for the test set (Table 1) within sea-ice zones 0 – 4. The table lists biases, standard deviations and RMS (all in K) for the performance metric, which is the SMAP V-pol specular surface TB bias ΔT_B,0_(SMAP V). Case 1 refers to the algorithm using the AMSR2 TOA as input. Case 2 refers to the algorithm using the AMSR2 measured minus expected specular surface emissivities as input. The estimated average g_ice_ values in the 2^nd^ column are obtained by dividing the values for the TB biases without correction in each zone by 125 K, which is a typical value for the L-band TB difference between sea-ice and ocean scenes.

		no correction			Case 1 using AMSR2 TB TOA			Case 2 using AMSR2 E0 meas - exp		
Zone	estimated g_ice_	Bias	Std.Dev	RMS	Bias	Std.Dev	RMS	Bias	Std.Dev	RMS
**0**	< 0.05%	0.07	0.20	0.21						
**1**	0.3%	0.33	0.28	0.43	−0.03	0.27	0.27	0.02	0.28	0.28
**2**	0.6%	0.70	0.56	0.89	0.06	0.52	0.52	0.02	0.50	0.50
**3**	2.2%	2.76	2.02	3.42	0.19	1.32	1.33	−0.10	1.33	1.33
**4**	9.6%	11.62	8.07	14.14	−0.01	3.12	3.12	−0.51	3.35	3.39

## Appendix A. Fisher Linear Discriminant Analysis

Our sea-ice detection algorithm is based on the Fisher Linear Discriminant Analysis, which is a well-established statistical technique in pattern classification for distinguishing two classes of data [38,39]. This appendix gives a brief summary of the method.

Consider two data sets Ω_i_, i = 1, 2, each of which contains N_i_ data points. Each data point itself is a measurement in a d-dimensional space and thus labelled as a d-dimensional vector **X**. In our case, the measurement vector **X** consists of the 10 AMSR2 TOA TB or the measured–expected specular surface emissivities (Table 2). The sample means **M**_**i**_ of each set Ω_i_ are given by:

(A1)
$$M_{\text{i}}=\frac{1}{{\text{N}}_{\text{i}}}\cdot \sum_{\text{X}\in {\text{Ω}}_{\text{i}}}\text{X}$$

which are both again d-dimensional vectors. The Fisher Discriminant Analysis considers the projection of the d-dimensional data vectors onto a fixed d-dimensional vector **W**. The projected means are thus:

(A2)
$${\tilde{\text{M}}}_{\text{i}}=\frac{1}{{\text{N}}_{\text{i}}}\cdot \sum_{\text{X}\in {\text{Ω}}_{\text{i}}}{\text{W}}^{\text{T}}\cdot \text{X}={\text{W}}^{\text{T}}\cdot M_{\text{i}}$$

where **W**^**T**^ denotes the transposed vector and thus **W**^**T**^ **X** is the scalar product between the two vectors **W** and **X**. The distance between the projected means is:

(A3)
$$\left|{\tilde{\text{M}}}_{1}-{\tilde{\text{M}}}_{2}\right|=\left|W^{\text{T}}\cdot \left(M_{1}-M_{2}\right)\right|.$$

The variances and scatters of each projected data set are:

(A4)
$${\tilde{\sigma }}_{\text{i}}^{2}=\frac{1}{{\text{N}}_{\text{i}}}\cdot \sum_{\text{X}\in {\text{Ω}}_{\text{i}}}{\left({\text{W}}^{\text{T}}\cdot \text{X}-{\tilde{\text{M}}}_{\text{i}}\right)}^{2}=:\frac{1}{{\text{N}}_{\text{i}}}\cdot {\tilde{\text{s}}}_{\text{i}}{}^{2}$$

and the variance of the combined (pooled) data is:

(A5)
$${\tilde{\sigma }}^{2}=\frac{1}{{\text{N}}_{1}+{\text{N}}_{2}}\cdot \left({\tilde{\text{s}}}_{1}^{2}+{\tilde{\text{s}}}_{2}^{2}\right).$$

The goal is to determine the projection vector **W** that leads to the maximum separation between the two data sets. This is achieved by maximizing the objective function J(**W**), which maximizes the distance between the projected data points by simultaneously minimizing the pooled projected variance:

(A6)
$$\text{J}(W)=\frac{\left|{\tilde{\text{M}}}_{1}-{\tilde{\text{M}}}_{2}\right|}{{\tilde{\text{s}}}_{1}^{2}+{\tilde{\text{s}}}_{2}^{2}}.$$

The optimization of J(**W**) employs the covariance and scatter matrices of the individual sets Ω_i_:

(A7)
$$\mathrm{Cov}\left({\text{Ω}}_{\text{i}}\right)=\frac{1}{{\text{N}}_{\text{i}}}\cdot \sum_{\text{X}\in {\text{Ω}}_{\text{i}}}\left(\text{X}-{\text{M}}_{\text{i}}\right)\cdot {\left(\text{X}-{\text{M}}_{\text{i}}\right)}^{\text{T}}=:\frac{1}{{\text{N}}_{\text{i}}}\cdot {\text{S}}_{\text{i}}$$

and the within-scatter matrix **S**:

(A8)
$$\text{S}={\text{S}}_{1}+{\text{S}}_{2}.$$

The optimized projection vector **W** is determined by the expression:

(A9)
$$W=S^{-1}\cdot \left(M_{1}-M_{2}\right)$$

and the value of the discriminant is given by ${\text{W}}^{\text{T}}\cdot \text{X}$. The normalization of the projection vector is arbitrary. In our study we have normalized it to have unit length, i.e., ${\text{W}}^{\text{T}}\cdot \text{W}=1$. In order to obtain the decision criterion between the two classes, one creates plots of the probability density functions pdf_i_ (i = 1, 2) of the discriminant values in each class (Figure 4). The standard way of deciding if an event falls into class 1 (class 2) is the check if the discriminant pdf of class 1 (class 2) is larger than the discriminant pdf for class 2 (class 1). That puts the value d for the decision boundary between the two classes at the abscissa of the intersection point between the two pdf. In the context of Figure 4:

(A10)
$$\begin{array}{l} \left({\text{W}}^{\text{T}}\cdot \text{X}\right)>\text{d}\Rightarrow {\text{pdf}}_{2}>{\text{pdf}}_{1}\Rightarrow \text{X}\in \;\text{Class}\;2\\ \left({\text{W}}^{\text{T}}\cdot \text{X}\right)<\;\text{d}\Rightarrow {\text{pdf}}_{1}>{\text{pdf}}_{2}\Rightarrow \text{X}\in \;\text{Class}\;1 \end{array}$$
